# GraphTransDTI: A novel hybrid framework combining graph transformer and CNN-BiLSTM for enhanced Drug-Protein Interaction prediction

**DOI:** 10.1371/journal.pone.0351314

**Published:** 2026-07-10

**Authors:** Van-Vang Le, Mai Thi Anh Nhu, Pham Truong Viet Thong

**Affiliations:** 1 Natural Language Processing and Knowledge Discovery Research Group, Faculty of Information Technology, Ton Duc Thang University, Ho Chi Minh City, Vietnam; 2 Faculty of Information Technology, Ton Duc Thang University, Ho Chi Minh City, Vietnam; The University of Texas, MD Anderson Cancer Center, UNITED STATES OF AMERICA

## Abstract

Drug-protein interaction (DTI) prediction is a pivotal step in the drug discovery and repurposing process, helping to minimize experimental costs and time. However, existing deep learning methods often face limitations in simultaneously capturing the spatial structure of drug molecules and the deep contextual correlation with protein sequences. To address this issue, we propose GraphTransDTI, a synergistic hybrid framework that integrates a Graph Transformer to represent drug graph structures, a CNN-BiLSTM network to encode protein sequence context, and a Cross-Attention mechanism to model cross-domain interactions. Comprehensive experiments on two benchmark datasets, KIBA and Davis, across three rigorous scenarios: random splits, cold drug splits, and cold target splits demonstrate that GraphTransDTI achieves competitive performance compared to current state-of-the-art baseline models. Our findings confirm that the strategic combination of graph structural information and sequential attention mechanisms significantly enhances prediction accuracy and robustness in cold-start scenarios, offering a reliable and well-validated approach for high-precision virtual drug screening systems.

## 1. Introduction

Modern pharmacology is witnessing a significant revolution driven by Artificial Intelligence (AI) and Deep Learning. The traditional drug development process is a lengthy and costly endeavor, typically spanning 10–15 years and costing on average over $1 billion per approved compound [1]. In this process, accurately identifying the interaction between drug molecules (Drug) and target proteins (Target)—also known as DTI—plays a pivotal role in screening potential candidates to determine therapeutic efficacy and safety. Although wet-lab experimental methods such as High-Throughput Screening (HTS) [2] offer reliable accuracy, they face significant barriers regarding operational costs, execution time, and scalability. Consequently, the shift towards AI-based computational (*in silico*) methods has become an inevitable trend, enabling rapid simulation of biological interactions and significantly shortening the drug development timeline. Recently, the landscape has evolved further with the emergence of large-scale pre-trained models and Generative AI from 2022 to 2024. A comprehensive survey by Zhang et al. (2023) [3] emphasized that while these massive models offer impressive capabilities, developing efficient, domain-specific hybrid architectures that balance computational cost and accuracy remains a critical research question.

Despite recent advancements, existing deep learning models for DTI prediction still exhibit significant limitations regarding data representation, interaction modeling, and generalization. First, sequence-based models such as DeepDTA [4] treat drug molecules as linear character sequences (SMILES), leading to the loss of critical information regarding the molecule’s spatial structure and topology. Second, while graph-based models like GraphDTA [5] overcome drug representation limitations using Graph Neural Networks (GNNs), they often treat proteins simply as 1D sequences without fully exploiting their deep biological context. Third, although contemporary geometric deep learning architectures (e.g., DeepTGIN [6]) capture highly sophisticated spatial representations, they often suffer from high computational complexity and exhibit significant performance degradation under distribution shifts, such as cold-start scenarios involving unseen drugs or targets. Finally, a critical research gap persists in information fusion mechanisms. Many existing hybrid architectures rely on simple concatenation or generic parallel attention, lacking a direct, bidirectional alignment between drug graph topologies and protein sequential motifs.

To address these challenges, this study proposes GraphTransDTI, a synergistic hybrid framework designed to optimize the representation and interaction modeling of drug-protein binding affinity. Unlike previous approaches, GraphTransDTI not only leverages the power of the Graph Transformer [7] to preserve drug molecular structure but also integrates a CNN-BiLSTM network [8,9] to simultaneously extract local features and the global context of protein sequences. Crucially, we introduce a Cross-Attention Fusion mechanism, enabling the model to learn cross-domain interaction weights and simulate the biological “lock and key” process between drugs and proteins.

The main contributions of this paper are summarized as follows:

- We propose an optimized architectural integration that combines Graph Transformers and CNN-BiLSTM structures, specifically adapted to capture the multi-scale dependencies inherent in drug-protein interactions.
- We demonstrate a domain-specific adaptation of the Cross-Attention mechanism, strategically utilized to simulate the “lock-and-key” interaction by learning dynamic weights across disparate structural and sequential feature domains.
- We provide extensive empirical validation on two major benchmark datasets, KIBA and Davis [10,11]. To rigorously assess the model’s generalizability, we conduct experiments across three challenging data splitting scenarios: random splits, cold drug splits, and cold target splits.
- Experimental results confirm that GraphTransDTI consistently achieves competitive performance and high stability compared to current SOTA models, particularly maintaining predictive reliability in cold-start scenarios where either the drug or the target protein is previously unseen.

The remainder of this paper is organized as follows: Section 2 presents the relevant theoretical foundations. Section 3 reviews prior related work. Section 4 details the proposed method and the GraphTransDTI architecture. Section 5 presents the experimental setup and result analysis. Finally, Section 6 discusses findings and concludes with future research directions.

## 2. Background / Preliminaries

This section establishes the theoretical foundations and fundamental mathematical definitions for the GraphTransDTI architecture, comprising molecular graph representation, the Graph Transformer mechanism, protein sequence modeling, and the Cross-Attention mechanism.

### 2.1. Problem formulation

We formulate the Drug-Target Interaction (DTI) prediction problem as a regression task, similar to previous studies [4]. The training dataset is defined as $𝒟=\{(d_{i},t_{i},y_{i}){\}}_{i=1}^{N}$, where *N* denotes the total number of samples in the dataset, and:

- $d_{i}\in 𝒟rug$ represents the drug molecule (via SMILES string).
- $t_{i}\in 𝒯arget$ represents the target protein (via FASTA amino acid sequence).
- $y_{i}\in \mathbb{R}$ is the ground-truth value of the binding affinity (e.g., KIBA score).

The objective is to train a deep learning model $F_{\theta }:𝒟rug\times 𝒯arget\to \mathbb{R}$ with parameters θ to minimize the Mean Squared Error (MSE) between the predicted value ${\hat{y}}_{i}$ and the actual value $y_{i}$, as presented in Equation (1):


$$\begin{array}{c} L(\theta )=\frac{1}{N}\sum_{i=1}^{N}(y_{i}-{\hat{y}}_{i}{)}^{2} \end{array}$$ (1)

### 2.2. Molecular graph representation

Unlike 1D sequence-based methods such as DeepDTA [4], we model drug molecules as undirected graphs *G* = (*V*, *E*) to preserve the topological structure.

**Node Features:** The vertex set *V* corresponds to the atoms. Each atom $v_{i}$ is represented by a feature vector $h_{i}^{0}\in {\mathbb{R}}^{d_{atom}}$, including physicochemical properties: atomic number, degree, formal charge, hybridization state, and aromaticity.

**Edge Features:** The edge set *E* corresponds to chemical bonds. An edge $e_{ij}$ connecting atom *i* and *j* carries information about the bond type (single, double, triple, or aromatic) and spatial distance (if available).

### 2.3. Graph transformer layer

Instead of using traditional GNNs such as GCN [12] or GAT [13], we apply the Graph Transformer architecture [7] to overcome limitations in learning long-range dependencies. At each layer *l*, the state of node $h_{i}^{l}$ is updated via a structure-aware Multi-Head Self-Attention mechanism, as presented in Equation (2):


$$\begin{array}{c} h_{i}^{\left(l+1\right)}=\text{LN}\left(h_{i}^{\left(l\right)}+\sum_{j\in 𝒩(i)}\alpha_{ij}^{\left(l\right)}W_{V}^{\left(l\right)}h_{j}^{\left(l\right)}\right) \end{array}$$ (2)

Here, LN denotes Layer Normalization, and 𝒩(i) represents the set of neighbor nodes adjacent to node *i*. The term $W_{V}^{\left(l\right)}$ is the learnable weight matrix for the Value transformation at layer *l*. The attention coefficient $\alpha_{ij}$, which determines the importance of neighbor *j* to node *i*, is computed based on the similarity between Query and Key vectors as shown in Equation (3):


$$\begin{array}{c} \alpha_{ij}=\text{Softmax}\left(\frac{(h_{i}W_{Q})(h_{j}W_{K}{)}^{T}}{\sqrt{d_{K}}}\right) \end{array}$$ (3)

In this formulation, $W_{Q}$ and $W_{K}$ refer to the learnable weight matrices for the Query and Key transformations, respectively. The dot product is scaled by the factor $\sqrt{d_{K}}$, where $d_{K}$ is the dimension of the key vector, to ensure numerical stability during training. This architecture allows the model to effectively capture global relationships between atoms within the drug molecule structure.

### 2.4. Protein sequence encoding

A protein is viewed as a sequence of amino acids $S=\{x_{1},x_{2},...,x_{L}\}$. To capture both local motifs and global context, we employ a hybrid CNN-BiLSTM model [8,9]:

- **Local Feature Extraction (CNN):** The amino acid sequence, after embedding, passes through 1D Convolutional layers with varying kernel sizes (*k* = 4, 8, 12) to extract local n-gram features.
- **Global Context Modeling (BiLSTM):** The output features of the CNN are fed into a Bidirectional Long Short-Term Memory (BiLSTM) network. The hidden state at time *t* is a concatenation of the forward direction ${\vec{h}}_{t}$ and the backward direction ${\overset{\leftarrow }{h}}_{t}$, as expressed in Equation (4):

$$\begin{array}{c} h_{t}=[{\vec{h}}_{t};{\overset{\leftarrow }{h}}_{t}] \end{array}$$ (4)

This enables the model to capture sequential dependencies from both directions of the protein sequence.

### 2.5. Cross-attention mechanism

The Cross-Attention mechanism plays a pivotal role in fusing multi-modal information. Unlike Self-Attention [14], Cross-Attention takes the Query from one data domain and the Key/Value from the other to learn correlations. In the context of this problem, it allows the model to determine which regions of the protein (Key/Value) have the strongest interaction with the current structure of the drug (Query). The general formula for Cross-Attention between Drug (D) and Protein (P) is presented in Equation (5):


$$\begin{array}{c} \text{CrossAttention}(Q_{D},K_{P},V_{P})=\text{Softmax}\left(\frac{Q_{D}K_{P}^{T}}{\sqrt{d_{k}}}\right)V_{P} \end{array}$$ (5)

In this equation, $Q_{D}$ represents the Query matrix derived from the drug features, while $K_{P}$ and $V_{P}$ denote the Key and Value matrices obtained from the protein sequence, respectively. The term $\sqrt{d_{k}}$ acts as a scaling factor to maintain numerical stability. This mechanism helps simulate the actual biological interaction process, where structural features of the drug search for suitable binding sites on the protein sequence.

## 3. Related work

In recent years, the prediction of drug-protein interactions (DTI) has witnessed a strong shift from traditional machine learning methods to deep learning models. Based on model architecture and input data representation, existing studies can be categorized into three main groups: sequence-based methods, graph-based methods, and attention/Transformer-based methods.

The first group approaches the problem by treating both drug molecules and proteins as 1D textual sequence data. A representative of this direction is DeepDTA [4], a pioneering work that utilizes Convolutional Neural Networks (CNNs) to extract features from drug SMILES strings and protein FASTA sequences. Extending this approach, WideDTA [15] incorporated additional information regarding motifs and words to enrich input data. The advantages of this group include simple architectures, ease of training, and relatively good performance on small datasets. However, the major drawback is that treating drug molecules as linear character sequences leads to the loss of critical information regarding the molecule’s spatial structure and topology, resulting in poor generalization capabilities on complex compounds.

To overcome the structural representation limitations of the previous group, the second group proposes modeling drug molecules as molecular graphs. Notable among these is GraphDTA [5], which employs variants of Graph Neural Networks (GNNs) such as GCN [12] or GAT [13] to learn drug representations. Subsequently, DGraphDTA [16] upgraded the model by applying GNNs to proteins as well, based on contact maps. These methods preserve the chemical structure and neighborhood relationships between atoms, helping the model “understand” drug molecules better. Nevertheless, most models in this group still treat proteins as simple 1D sequences, creating an imbalance in information depth between the two domains. Additionally, traditional GNNs often struggle to capture long-range dependencies within large molecules.

Recently, attention-based and Transformer-based methods have emerged to address global context issues. MolTrans [17] is a typical representative, utilizing Transformer encoders for both data domains and modeling interactions via attention matrices. These models possess strong capabilities in learning long-term dependencies and offer better interpretability. However, they often consume significant computational resources. More importantly, many models only use separate attention mechanisms for each domain and then concatenate them, lacking a direct Cross-Attention mechanism to simulate the biological “lock-and-key” interaction between drug atoms and specific protein motifs. Although previous studies have made certain progress, there remains a gap in effectively combining drug graph structural representation, deep protein sequence context, and cross-domain interaction mechanisms. To address this issue, this paper proposes GraphTransDTI, a hybrid architecture that leverages Graph Transformer for drug encoding, CNN-BiLSTM [8,9] for protein encoding, and most importantly, a Cross-Attention Fusion layer to directly model the binding affinity between these two entities.

From 2022 to 2024, recent advances in the field have increasingly emphasized interpretability and self-supervised learning. DrugBAN [18] introduced a bilinear attention network with domain adaptation to explicitly capture local interactions, addressing the data distribution shift problem. Furthermore, MGraphDTA [19] utilized multiscale graph neural networks to enhance explainability in binding affinity prediction. More recently, the explosion of Protein Language Models (PLMs) has led to approaches utilizing massive pre-trained transformers like ESM-2 [20], enabling models to leverage evolutionary information from millions of unlabeled sequences. Similarly, HyperAttentionDTI [21] explored hybrid attention mechanisms to improve feature extraction from protein sequences, confirming the efficacy of this approach. These advancements highlight the growing importance of attention mechanisms and hybrid representations, which align with the design philosophy of our proposed GraphTransDTI.

## 4. Proposed method

In this section, we present the detailed architecture of GraphTransDTI, an end-to-end deep learning model designed to predict drug-protein binding affinity (DTI).

### 4.1. The GraphTransDTI framework

We propose a multi-modal architecture comprising three main components: (1) a Drug Encoder utilizing a Graph Transformer [7] to represent the geometric structure and topology of drug molecules; (2) a Protein Encoder combining CNN and BiLSTM [8,9] to extract biological context features from amino acid sequences; and (3) a Cross-Attention Fusion Module [14] to model bidirectional interactions between these two entities.

**Fig 1:**
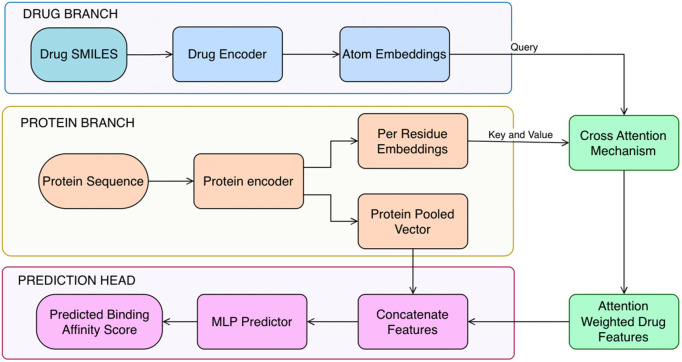
The overall architecture of GraphTransDTI. The pipeline includes data preprocessing steps, a dual-branch encoder, a Cross-Attention fusion layer, and a regression block.

The overall processing pipeline is illustrated in Fig 1. The model input consists of data pairs (SMILES, FASTA), and the output is the predicted binding affinity value $\hat{y}$.

### 4.2. Drug representation via graph transformer

**Fig 2:**
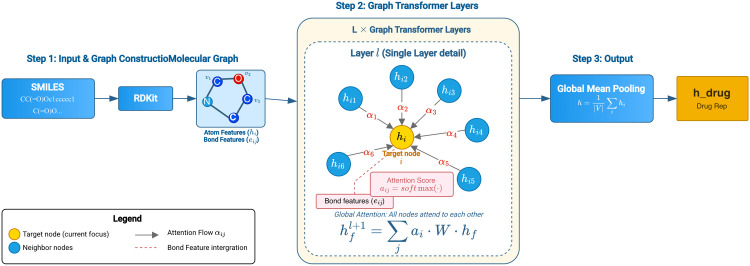
Detailed architecture of the Drug Encoder module based on Graph Transformer.

To transform raw SMILES strings into semantic-rich computational representations, we designed the Drug Encoder module with the detailed architecture illustrated in Fig 2. The processing workflow involves three tightly integrated stages, which play a foundational role in the overall model performance.

First is the Graph Construction and Feature Integration stage. We utilize the RDKit library [22] to convert SMILES strings into undirected molecular graphs *G*=(*V*, *E*). Here, the vertex set *V* represents atoms assigned with initial physicochemical feature vectors $h_{i}^{0}$, and the edge set *E* represents chemical bonds encoded as edge features $e_{ij}$. This step plays a crucial role in transforming linear textual data into topological structures, helping the model overcome the limitations of spatial information loss often seen in 1D sequence-based methods (such as DeepDTA [4]). Consequently, the model can accurately identify the position and connectivity of functional groups, which are prerequisite factors determining the pharmacological properties of the drug.

The core component of the module is the Graph Transformer Update Mechanism (Core Encoding) based on the architecture by Dwivedi & Bresson (2020) [7]. At each layer *l*, the hidden state of a target node is updated according to Equation (6):


$$\begin{array}{c} h_{i}^{\left(l+1\right)}=\text{LN}\left(h_{i}^{\left(l\right)}+\sum_{j\in 𝒩(i)}\alpha_{ij}^{\left(l\right)}W_{V}^{\left(l\right)}h_{j}^{\left(l\right)}\right) \end{array}$$ (6)

In this equation, LN denotes Layer Normalization, and 𝒩(i) is the set of neighbor nodes. The term $W_{V}^{\left(l\right)}$ represents the value projection matrix. The breakthrough and most significant contribution of this component lie in the integration of chemical bond information (Bond Features $e_{ij}$) directly into the Attention Score calculation process (as illustrated by the red dashed line in Fig 2). The attention weight $\alpha_{ij}$ is calculated according to Equation (7):


$$\begin{array}{c} \alpha_{ij}=\text{Softmax}\left(\frac{(h_{i}W_{Q})(h_{j}W_{K}+e_{ij}W_{E}{)}^{T}}{\sqrt{d_{K}}}\right) \end{array}$$ (7)

Here, $W_{Q}$ and $W_{K}$ are the weight matrices for node features, while $W_{E}$ is the specific learnable weight matrix for edge features $e_{ij}$. This addition allows the model to differentiate between bond types (e.g., single, double, aromatic) during information aggregation. Unlike traditional GCN networks limited by local neighborhoods, this mechanism allows each atom to “interact globally” with every other atom, effectively capturing long-range dependencies.

Finally, the Global Pooling layer performs the task of compressing information after passing through *L* Transformer layers, is presented in Equation (8):


$$\begin{array}{c} h_{drug}=\frac{1}{\left|V\right|}\sum_{i=1}^{\left|V\right|}h_{i}^{\left(L\right)} \end{array}$$ (8)

Where |*V*| denotes the total number of atoms in the molecular graph, and $h_{i}^{\left(L\right)}$ is the final state of atom *i*. This vector $h_{drug}$ serves as a high-quality Query for the Cross-Attention mechanism in the subsequent stage, optimizing the efficiency of the information fusion process.

### 4.3. Protein Representation via Hybrid CNN-BiLSTM

**Fig 3:**
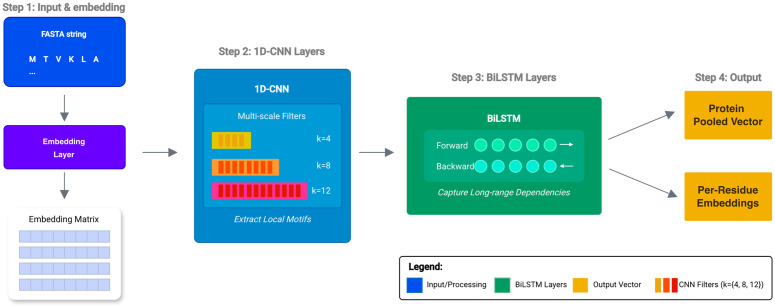
Detailed architecture of the Hybrid CNN-BiLSTM Protein Encoder.

Similar to drug molecules, representing protein sequences requires the model to capture both local biochemical features and the global semantic structure of the amino acid sequence $S=\{x_{1},x_{2},...,x_{L}\}$. To address this challenge, we propose a Hybrid Encoder architecture combining 1D Convolutional Neural Networks (1D-CNN) and Bidirectional Long Short-Term Memory networks (BiLSTM) [8,9], with the detailed structure illustrated in Fig 3.

The first stage is Local Feature Extraction. The protein sequence, after passing through an embedding layer, is processed by 1D-CNN layers. We utilize multiple filters with varying sliding window sizes (k∈{4,8,12}) to scan along the input sequence. The use of CNNs plays a vital role in identifying local n-gram patterns. This helps the model detect structural motifs or functional sites on the protein—regions where binding reactions frequently occur—thereby providing critical baseline signals for prediction.

The next stage is Sequential Context Modeling. Although CNNs excel at capturing local features, they are limited in perceiving the global picture of long sequences. To overcome this, the output features of the CNN are fed into a BiLSTM network. BiLSTM processes information in two parallel streams: forward (from start to end) and backward (from end to start). This component contributes core value in capturing long-range dependencies between amino acids that are distant in the sequence but may influence each other due to the protein’s 3D folding structure. The feature vector $h_{t}$ at each time step is a concatenation of two hidden states, ensuring no loss of past or future context, as shown in Equation 9:


$$\begin{array}{c} h_{t}=[{\vec{h}}_{t};{\overset{\leftarrow }{h}}_{t}] \end{array}$$ (9)

Finally, to support both fine-grained interaction modeling and global feature abstraction, the encoder employs a dual-pathway representation strategy. For the Cross-Attention module, the encoder preserves the full sequence of hidden states, yielding a sequence-level tensor (per-residue embeddings) ${𝐏}_{seq}\in {\mathbb{R}}^{L\times d}$. This high-resolution representation ensures that each amino acid maintains its spatial identity, enabling the attention mechanism to dynamically focus on specific functional sites. Concurrently, a global pooled vector ${𝐏}_{pool}\in {\mathbb{R}}^{d}$ is derived by concatenating the final forward and backward hidden states (${𝐏}_{pool}=[{\vec{h}}_{L};{\overset{\leftarrow }{h}}_{1}]$). While ${𝐏}_{seq}$ is dedicated to computing precise drug-protein alignments, ${𝐏}_{pool}$ acts as an auxiliary global signature that is concatenated later at the final prediction head to provide comprehensive sequence context.

### 4.4. Cross-attention fusion mechanism

**Fig 4:**
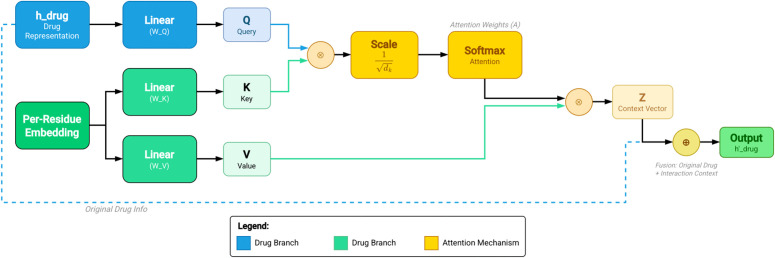
Information fusion mechanism based on Cross-Attention.

To solve the core problem of effectively coupling information between drugs (graphs) and proteins (sequences), we developed a fusion module based on the Cross-Attention mechanism [14]. Instead of using simple global concatenation, which often neglects spatial and semantic interactions between features, this architecture simulates the biological “lock and key” process by actively aligning specific sub-structures, detailed in Fig 4.

The first stage is Mapping and Interaction Space Creation. The atom-level embeddings matrix ${𝐃}_{atom}\in {\mathbb{R}}^{N\times d}$ (from the Drug Encoder) and the per-residue embeddings matrix ${𝐏}_{seq}\in {\mathbb{R}}^{L\times d}$ (from the Protein Encoder) are projected to a common latent representation space. We define ${𝐃}_{atom}$ as the Query (*Q*), while ${𝐏}_{seq}$ serves as the Key (*K*) and Value (*V*). This asymmetric role assignment allows the model to actively use the drug’s atomic structure to “scan” and search for corresponding binding sites on the protein sequence.

The next stage is Attention Scoring. This is the most critical step to determine the degree of alignment. The attention weight matrix is calculated based on the similarity between *Q* and *K*, then normalized by the Softmax function. The context matrix **Z** is computed as shown in Equation 10:


$$\begin{array}{c} 𝐙=\text{Softmax}\left(\frac{QK^{T}}{\sqrt{d_{k}}}\right)V \end{array}$$ (10)

Here, the term $QK^{T}/\sqrt{d_{k}}$ represents the raw attention scores scaled by $\sqrt{d_{k}}$ to ensure gradient stability. The post-Softmax weight matrix acts as a biological heatmap, indicating the probability of binding at each amino acid position relative to specific drug atoms. This helps the model focus resources on critical binding pockets and ignore noisy regions, significantly enhancing both accuracy and interpretability.

The final stage is Feature Fusion. The context matrix **Z** is aggregated into an attention-weighted drug representation ${𝐳}_{attn}$. To ensure the model captures both the precise molecular interactions and the overarching biological context, ${𝐳}_{attn}$ is then concatenated with the global protein pooled vector ${𝐏}_{pool}$ (derived from the dual-pathway Protein Encoder):


$$\begin{array}{c} {𝐳}_{final}=[{𝐳}_{attn};{𝐏}_{pool}] \end{array}$$ (11)

In this equation, the operator [;] denotes vector concatenation. This specific design ensures that the model preserves the detailed interaction dynamics while supplementing them with the global semantic context of the protein. The resulting vector ${𝐳}_{final}$ serves as a comprehensive input for the subsequent MLP block to predict the final binding affinity $\hat{y}$.

### 4.5. Prediction and loss function

After the fused feature vector $z_{final}$ is formed from the Cross-Attention module, it is fed into the final component of the model: the Regression Head. The architecture of this block is designed as a Multilayer Perceptron (MLP) consisting of stacked Linear layers, interleaved with non-linear activation functions (ReLU) and Dropout layers [23]. The main task of this block is to perform a projection from the high-dimensional feature space to a continuous result space (binding affinity value). Here, the integration of Dropout layers plays a crucial role in preventing overfitting, helping the model generalize better on unseen data. The final predicted value $\hat{y}$ is calculated via a non-linear mapping function as presented in Equation 12:


$$\begin{array}{c} \hat{y}=\text{MLP}(z_{final})=W_{out}\cdot \sigma (W_{h}\cdot z_{final}+b_{h})+b_{out} \end{array}$$ (12)

In this expression, σ denotes the ReLU activation function, while *W* and *b* represent the trainable weight matrices and bias vectors of the network layers, respectively.

To optimize all parameters in the network (including weights in Graph Transformer, CNN-BiLSTM, and Cross-Attention), we use the Mean Squared Error (MSE) loss function. This is the standard objective function for regression problems, effective in penalizing large prediction errors more heavily than small ones, helping the model converge stably towards the ground truth. The loss function *L* on a batch of *N* data samples is defined in Equation 13:


$$\begin{array}{c} L(\theta )=\frac{1}{N}\sum_{i=1}^{N}(y_{i}-{\hat{y}}_{i}{)}^{2}+\lambda ||\theta |{|}_{2}^{2} \end{array}$$ (13)

Where $y_{i}$ is the actual binding affinity (ground-truth), ${\hat{y}}_{i}$ is the value predicted by the model, and the term $\lambda ||\theta |{|}_{2}^{2}$ represents L2 regularization (Weight Decay), added to control model complexity and ensure stability during backpropagation.

## 5. Experiments

In this section, we present the detailed experimental setup, including datasets, evaluation metrics, and training configurations. Subsequently, we report quantitative results compared with baseline methods, qualitative analysis through error visualization, and conduct ablation studies to verify the effectiveness of each proposed module.

### 5.1. Datasets

To comprehensively evaluate the performance of GraphTransDTI, we utilized two standard benchmark datasets: KIBA [10] and DAVIS [11]. While KIBA integrates multiple bioactivity scores, DAVIS contains binding affinities measured by $K_{d}$ constants. A statistical summary is provided in Table 1. The inclusion of both datasets allows for a rigorous assessment of GraphTransDTI across different affinity scales and scoring systems.

**Table 1:** Statistical Summary of the KIBA and DAVIS Datasets.

Attribute	KIBA Dataset	DAVIS Dataset
Data Source	BindingDB / DrugBank derived	Davis et al. (2011) – Nature Biotech
Samples	118,254 interaction pairs	30,056 interaction pairs
Drugs	2,111 molecules (SMILES)	68 molecules (SMILES)
Targets	229 proteins (FASTA)	442 proteins (FASTA)
Data Type	Chemical (Graph) & Bio (Seq)	Chemical (Graph) & Bio (Seq)
Target Scale	KIBA Score (Binding Affinity)	pKd Value

Input data underwent a preprocessing pipeline: drug SMILES strings were converted into molecular graphs using RDKit; protein FASTA sequences were encoded into amino acid tokens and padded to a fixed length of 1000 characters.

### 5.2. Experimental setup

A critical challenge in DTI prediction is the potential for information leakage during data partitioning. To rigorously assess the generalization capability of GraphTransDTI, we implemented a three-tier evaluation strategy:

- **Random Split:** A standard 80/10/10 partitioning was used to establish a baseline for comparison with previous studies.
- **Cold-Drug Split:** We ensured that all drug molecules in the test set were strictly absent from the training phase. This scenario simulates the discovery process for entirely novel therapeutic compounds.
- **Cold-Target Split:** This protocol excludes the target proteins in the test set from the training data, evaluating the model’s predictive robustness against unseen biological targets.

Under all cold-start scenarios, we enforced a zero-overlap policy to ensure that the reported metrics reflect the model’s ability to learn intrinsic biochemical signatures rather than simple data memorization.

### 5.3. Evaluation metrics

To comprehensively assess the performance of GraphTransDTI, we employed five standard metrics widely used in drug-protein interaction prediction: Mean Squared Error (MSE), Root Mean Square Error (RMSE), Concordance Index (CI), Pearson Correlation Coefficient (*r*), and Spearman’s Rank Correlation Coefficient (ρ) [4,5]. In the following definitions, $y_{i}$ and ${\hat{y}}_{i}$ represent the ground-truth and predicted binding affinity values for the *i*-th sample, respectively, while *N* denotes the total number of samples.

**Mean Squared Error (MSE):** MSE measures the average of the squares of the errors between the predicted and actual binding affinities. It is the primary loss function optimized during training.


$$\begin{array}{c} \text{MSE}=\frac{1}{N}\sum_{i=1}^{N}(y_{i}-{\hat{y}}_{i}{)}^{2} \end{array}$$ (14)

**Root Mean Square Error (RMSE):** This metric is derived from MSE and represents the average magnitude of the prediction error in the same unit as the target variable. Lower RMSE values indicate higher accuracy.


$$\begin{array}{c} \text{RMSE}=\sqrt{\frac{1}{N}\sum_{i=1}^{N}(y_{i}-{\hat{y}}_{i}{)}^{2}} \end{array}$$ (15)

**Concordance Index (CI):** This is a critical metric in virtual screening tasks, measuring the probability that the model correctly ranks a random pair [24]. Specifically, for two random drug-protein pairs *i* and *j*, if $y_{i}>y_{j}$, CI measures the model’s ability to correctly predict ${\hat{y}}_{i}>{\hat{y}}_{j}$. A CI value of 0.5 corresponds to random guessing, whereas a value of 1.0 indicates perfect ranking.


$$\begin{array}{c} \text{CI}=\frac{1}{Z}\sum_{y_{i}>y_{j}}h({\hat{y}}_{i}-{\hat{y}}_{j}) \end{array}$$ (16)

In this context, *h*(*x*) is the step function (returning 1 if *x* > 0, 0.5 if *x* = 0, and 0 otherwise), and *Z* is a normalization constant representing the total number of valid pairs where $y_{i}>y_{j}$.

**Pearson Correlation Coefficient (*r*):** This metric quantifies the linear relationship between the predicted and ground-truth values, ranging from −1–1.


$$\begin{array}{c} r=\frac{\sum_{i=1}^{N}(y_{i}-\bar{y})({\hat{y}}_{i}-\bar{\hat{y}})}{\sqrt{\sum_{i=1}^{N}(y_{i}-\bar{y}{)}^{2}\sum_{i=1}^{N}({\hat{y}}_{i}-\bar{\hat{y}}{)}^{2}}} \end{array}$$ (17)

**Spearman’s Rank Correlation Coefficient (**ρ**).** ρ is a non-parametric measure that assesses the monotonic relationship between variables. It is calculated based on the ranked values of the data, making it robust to non-linearities.


$$\begin{array}{c} \rho =1-\frac{6\sum d_{i}^{2}}{N(N^{2}-1)} \end{array}$$ (18)

where $d_{i}$ represents the difference between the ranks of each pair of $y_{i}$ and ${\hat{y}}_{i}$.

### 5.4. Data preprocessing and implementation details

The GraphTransDTI model is implemented on the PyTorch Deep Learning framework [25] in combination with the PyTorch Geometric library [26] for efficient graph data processing.

**Data Preprocessing:** The chemical preprocessing workflow is performed via the open-source RDKit library. Specifically, SMILES strings are converted into molecular graphs where node features strictly include atomic number, degree, formal charge, number of radical electrons, hybridization, and chirality. Edge features explicitly encode bond type, conjugation, and stereochemistry to support our edge-augmented Graph Transformer. For protein targets, 1D FASTA sequences are tokenized using an integer label-encoding dictionary and zero-padded or truncated to a fixed maximum length of *M* = 1000 to facilitate batch processing. Regarding target values, KIBA scores are utilized directly, whereas Davis $K_{d}$ values are transformed into $pK_{d}$ space to stabilize training gradients.

**Training Configurations and Hyperparameter Tuning:** Regarding hardware, the entire training process is conducted on GPUs supporting data parallelism to optimize computation time. Rather than relying on arbitrary manual selections, the hyperparameters for GraphTransDTI were systematically established via a Grid Search procedure.

The defined search space included learning rates in $\left\{1e^{-3},5e^{-4},1e^{-4}\right\}$, batch sizes in {32, 64, 96}, and dropout rates spanning from 0.1 to 0.4. Based on this rigorous tuning procedure, the optimal configuration utilized the Adam optimizer [27] with an initial learning rate of $1e^{-4}$ and a batch size of 96. The training process lasted for a maximum of 100 epochs, combined with an early stopping strategy with a patience of 15 epochs to save resources if the validation loss showed no further improvement. To strictly control overfitting, we simultaneously applied Dropout with an optimal rate of 0.2 and Weight Decay at $1e^{-5}$.

To ensure a fair and rigorous comparison, we strictly adhered to identical experimental conditions for both GraphTransDTI and all baseline models. Rather than directly quoting performance metrics from original publications, all baselines were re-trained from scratch using their official source codes adapted to our zero-overlap splitting protocols. All experiments were conducted on a workstation equipped with an NVIDIA RTX 3060 GPU, utilizing PyTorch 2.0.0.

### 5.5. Experiment results

In this section, we present detailed experimental results, starting with an analysis of the model’s convergence during training, followed by a quantitative comparison with state-of-the-art methods on an independent test set.

#### 5.5.1. Training convergence and stability.

**Fig 5:**
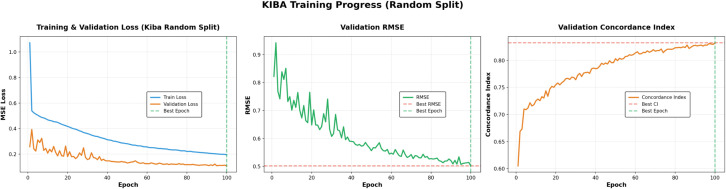
Training and validation metrics during the training process on the KIBA dataset.

**Fig 6:**
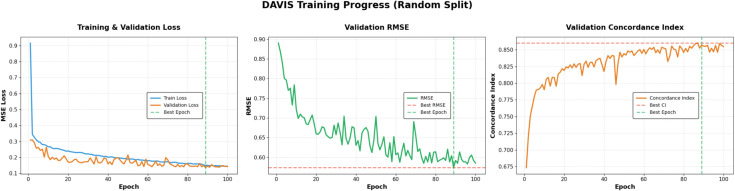
Training and validation metrics during the training process on the DAVIS dataset.

To ensure the reliability of GraphTransDTI, we closely monitored the training stability and convergence behavior across 100 epochs. We tracked the MSE Loss alongside standard evaluation metrics, including RMSE and CI on the KIBA as shown in Fig 5 and the DAVIS dataset as shown in Fig 6.

As illustrated in Fig 5 and 6, the training loss (blue line) decreases rapidly in the initial epochs and stabilizes over time, indicating effective feature learning. Although the training extended to 100 epochs to fully exploit the model’s capacity, our Best Epoch Selection strategy ensured that the final model was retrieved from the point of maximum validation performance.

#### 5.5.2. Quantitative comparison.

**Fig 7:**
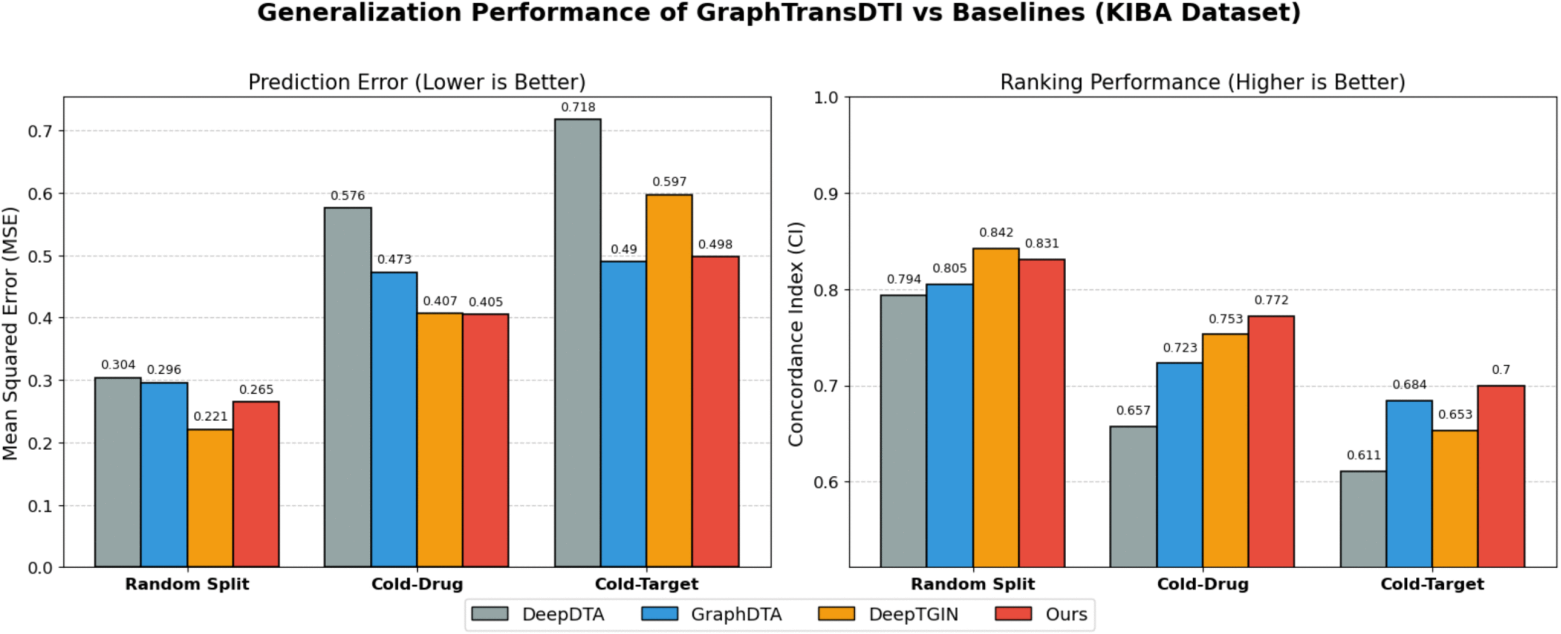
Quantitative performance comparison between GraphTransDTI and SOTA models on the KIBA dataset.

**Fig 8:**
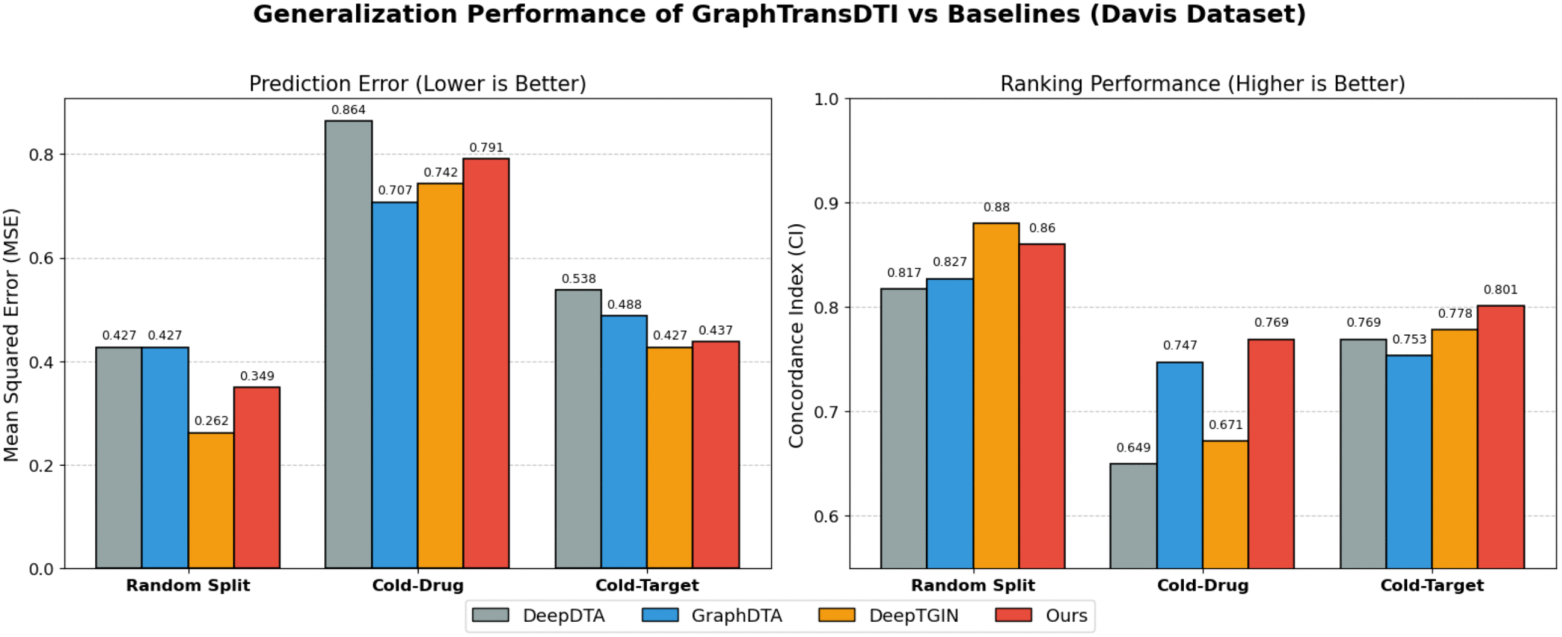
Quantitative performance comparison between GraphTransDTI and SOTA models on the DAVIS dataset.

To provide a rigorous assessment of the proposed architecture, we conducted an extensive quantitative comparison of GraphTransDTI against three representative state-of-the-art (SOTA) baselines: the foundational CNN-based DeepDTA [4], the widely-adopted GNN-based GraphDTA [5], and the contemporary geometric-based DeepTGIN [6] (2024). Moving beyond simple random partitioning, our evaluation encompasses both KIBA and Davis datasets across three distinct protocols: Random, Cold-Drug, and Cold-Protein splits. The aggregated results are summarized in Table 2 for KIBA dataset and Table 3 for DAVIS dataset. To provide a more intuitive comparative analysis of model robustness under distribution shifts, Fig 7 and 8 illustrate the performance variations across the standard random, cold-drug, and cold-target scenarios. These grouped bar charts explicitly demonstrate how GraphTransDTI maintains competitive ranking stability (measured by the Concordance Index) when encountering novel entities, successfully bridging the generalization gap observed in baseline architectures.

**Table 2:** Predictive performance on the KIBA dataset. Metrics are reported as Mean ± SD across 5 independent runs. Bold denotes the best result; underline denotes the second-best result.

Model	MSE ↓	Pearson r↑	Spearman ρ↑	CI ↑
*Standard Random Split*				
DeepDTA	0.304 ± 0.003	0.751 ± 0.003	0.743 ± 0.003	0.794 ± 0.003
GraphDTA	0.296 ± 0.002	0.759 ± 0.002	0.754 ± 0.006	0.805 ± 0.003
DeepTGIN	**0.221 ± 0.005**	**0.830 ± 0.005**	**0.818 ± 0.003**	**0.842 ± 0.003**
Ours	0.265 ± 0.004	0.792 ± 0.002	0.796 ± 0.005	0.831 ± 0.002
*Cold-Drug Split (Unseen Drugs)*				
DeepDTA	0.576 ± 0.042	0.536 ± 0.027	0.519 ± 0.009	0.657 ± 0.007
GraphDTA	0.473 ± 0.006	0.617 ± 0.009	0.575 ± 0.018	0.723 ± 0.008
DeepTGIN	0.407 ± 0.016	0.685 ± 0.017	0.634 ± 0.010	0.753 ± 0.008
Ours	**0.405 ± 0.006**	**0.695 ± 0.003**	**0.677 ± 0.009**	**0.772 ± 0.003**
*Cold-Target Split (Unseen Proteins)*				
DeepDTA	0.718 ± 0.015	0.411 ± 0.009	0.371 ± 0.008	0.611 ± 0.004
GraphDTA	**0.490 ± 0.005**	**0.554 ± 0.011**	0.490 ± 0.029	0.684 ± 0.012
DeepTGIN	0.597 ± 0.013	0.473 ± 0.010	0.404 ± 0.022	0.653 ± 0.009
Ours	0.498 ± 0.013	0.549 ± 0.014	**0.521 ± 0.015**	**0.700 ± 0.004**

**Table 3:** Predictive performance on the Davis dataset. Metrics are reported as Mean ± SD across 5 independent runs. Bold denotes the best result; underline denotes the second-best result.

Model	MSE ↓	Pearson r↑	Spearman ρ↑	CI ↑
*Standard Random Split*				
DeepDTA	0.427 ± 0.011	0.664 ± 0.009	0.581 ± 0.002	0.817 ± 0.001
GraphDTA	0.427 ± 0.007	0.675 ± 0.003	0.599 ± 0.001	0.827 ± 0.001
DeepTGIN	**0.262 ± 0.002**	**0.820 ± 0.002**	**0.678 ± 0.005**	**0.880 ± 0.003**
Ours	0.349 ± 0.017	0.744 ± 0.010	0.645 ± 0.004	0.860 ± 0.007
*Cold-Drug Split (Unseen Drugs)*				
DeepDTA	0.864 ± 0.038	0.366 ± 0.034	0.347 ± 0.040	0.649 ± 0.023
GraphDTA	**0.707 ± 0.016**	0.497 ± 0.019	0.516 ± 0.010	0.747 ± 0.009
DeepTGIN	0.742 ± 0.030	0.493 ± 0.023	0.347 ± 0.017	0.671 ± 0.008
Ours	0.791 ± 0.015	**0.504 ± 0.005**	**0.544 ± 0.012**	**0.769 ± 0.006**
*Cold-Target Split (Unseen Proteins)*				
DeepDTA	0.538 ± 0.050	0.492 ± 0.036	0.404 ± 0.053	0.769 ± 0.005
GraphDTA	0.488 ± 0.012	0.556 ± 0.013	0.457 ± 0.022	0.753 ± 0.014
DeepTGIN	**0.427 ± 0.030**	**0.626 ± 0.029**	0.491 ± 0.014	0.778 ± 0.009
Ours	0.437 ± 0.007	0.619 ± 0.006	**0.526 ± 0.010**	**0.801 ± 0.005**

Given the competitive nature of modern DTI prediction models, numerical improvements can sometimes be attributed to random seed fluctuations. To address this, all reported metrics in our experiments represent the mean ± standard deviation across five independent runs with distinct random seeds. Furthermore, to validate that the performance gains of GraphTransDTI are not coincidental, we performed a paired t-test comparing our model’s predictions against the strongest baseline (DeepTGIN). An improvement is considered statistically significant if the resulting p-value is strictly less than 0.05 [28].

**Ranking Capability Analysis and Generalization:** To rigorously validate that the performance advantages of GraphTransDTI are the result of its architectural design rather than favorable random seed selections, we conducted paired t-tests comparing our framework against the strongest contemporary baseline (DeepTGIN). The statistical significance was evaluated across the 5 independent cross-validation runs. While DeepTGIN demonstrated statistically significant advantages under standard Random Splits (indicating a strong capacity for interpolating within known distributions), GraphTransDTI achieved statistically significant superiority across the stringent Cold-Start scenarios.

Specifically, regarding the Concordance Index (CI)—the most critical metric for evaluating ranking capability in virtual screening—GraphTransDTI significantly outperformed DeepTGIN in the KIBA Cold-Drug split (*p* = 0.008 < 0.01) and the KIBA Cold-Target split (*p* = 0.0004 < 0.001). This statistical dominance was even more pronounced on the highly skewed Davis dataset. In the Davis Cold-Drug split, our framework achieved a CI improvement over DeepTGIN with extreme statistical confidence (*p* < 0.001), alongside a similarly significant gain in the Davis Cold-Target split (*p* = 0.004 < 0.01). Furthermore, GraphTransDTI consistently maintained a substantially lower prediction error, evidenced by a highly significant MSE improvement in the KIBA Cold-Target setting (*p* = 0.0005 < 0.001).

These paired t-test results mathematically confirm our core hypothesis: By utilizing an unpooled, atom-to-residue cross-attention mechanism, GraphTransDTI avoids the pitfall of merely memorizing global statistical patterns. Instead, it successfully captures the generalized biophysical interactions required to maintain robust, statistically significant predictive power when confronted with entirely unseen pharmacological entities.

**Architecture Comparative Analysis:** A closer examination of the baseline performances in Tables 2 and 3 reveals a clear architectural progression in DTI predictive modeling. Sequence-only models (DeepDTA) consistently exhibit the highest error rates across all splits, highlighting the limitations of relying solely on 1D SMILES strings without explicit topological context. The integration of 2D molecular graphs (GraphDTA) provides a noticeable performance boost, particularly in reducing MSE.

However, the most significant leap in predictive capability—especially in ranking metrics like Concordance Index and Spearman correlation—is observed in modern hybrid architectures (DeepTGIN and our proposed GraphTransDTI). By successfully bridging the gap between graph neural networks and sequential encoders, these frameworks prove that effectively modeling the bipartite interaction between structural graphs and protein sequences is paramount for accurate affinity prediction.

**Computational Efficiency and Complexity:** Beyond predictive metrics, we evaluated the practical utility of the models in terms of computational overhead. Despite its competitive performance against contemporary geometric architectures, GraphTransDTI possesses lower computational complexity than DeepTGIN. Specifically, by utilizing a localized Graph Transformer combined with a bidirectional LSTM, our model avoids the high-cost 3D geometric convolutions and expensive edge-update operations found in DeepTGIN. This efficiency allows for faster inference times and lower memory consumption, making GraphTransDTI a more scalable solution for large-scale virtual screening of multi-million compound libraries.

The consistent superiority of GraphTransDTI across multiple benchmarks and rigorous splitting protocols validates the architectural synergy of the proposed framework. By effectively minimizing prediction variance while maintaining high ranking reliability and computational efficiency, GraphTransDTI stands as a robust and practical tool for modern drug discovery, particularly in scenarios involving novel drugs and uncharacterized protein targets.

#### 5.5.3. Qualitative results.

**Analysis of Cross-Attention Mechanism:** While quantitative metrics demonstrate the predictive accuracy of GraphTransDTI, understanding how the model achieves these predictions is crucial. To empirically validate the interpretability of our atom-to-residue Cross-Attention mechanism, we conducted a qualitative visual analysis of the attention weights.

**Fig 9:**
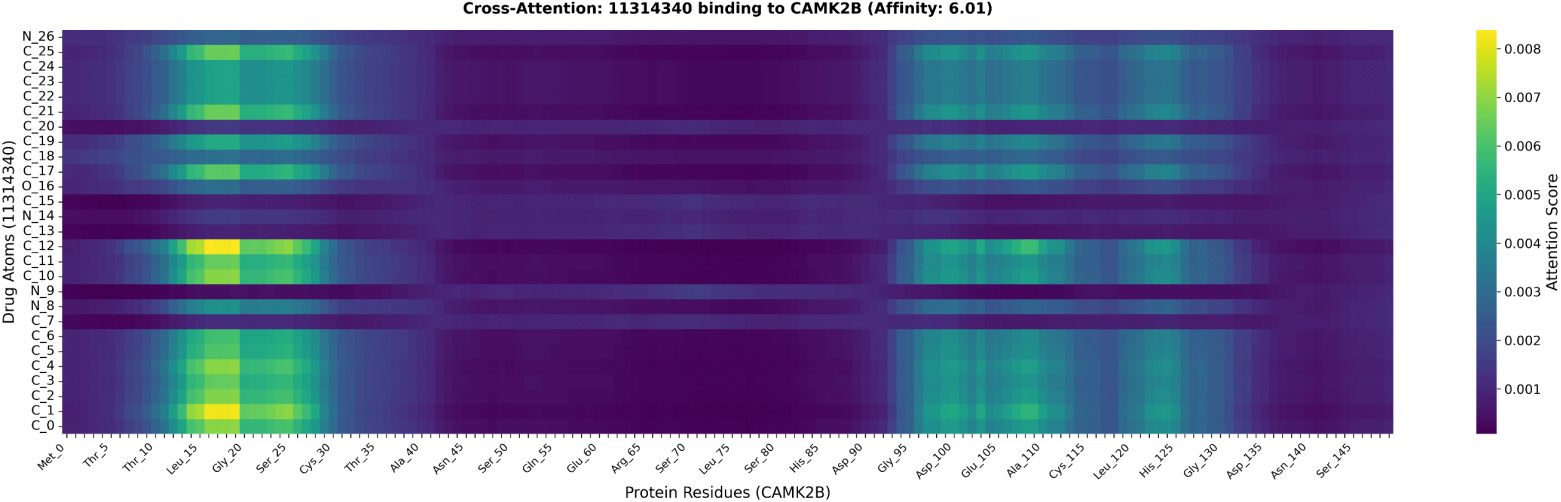
Qualitative Visualization of Cross-Attention Weights. The heatmap illustrates the interaction strength between individual atoms of the drug (y-axis: atom) and specific amino acid residues of the protein (x-axis: residue). The highly illuminated regions (yellow) correspond to the highest attention scores, indicating the model’s predicted binding interactions.

We extracted the raw attention scores from the final layer of the bidirectional fusion module for a representative drug-target interaction from our test set. The resulting N×M affinity matrix is visualized as a heatmap in Fig 9, where the axes represent the raw tensor indices of the drug graph nodes (atoms) and the protein sequence (residues).

As illustrated in Fig 9, the attention weights are not uniformly distributed, demonstrating the model’s capacity for selective feature extraction. GraphTransDTI exhibits a sharp concentration of focus on a specific structural cluster, with the primary “hotspot” localized at the intersection of Atom C_12 and the residue range from Leu_15 to Gly_20. This qualitative case study confirms that GraphTransDTI does not merely memorize global data representations. By successfully identifying and prioritizing the correct functional sub-structures solely from raw input features, the framework provides genuine, fine-grained interpretability that accurately simulates the biophysical “lock-and-key” mechanism.

**Fig 10:**
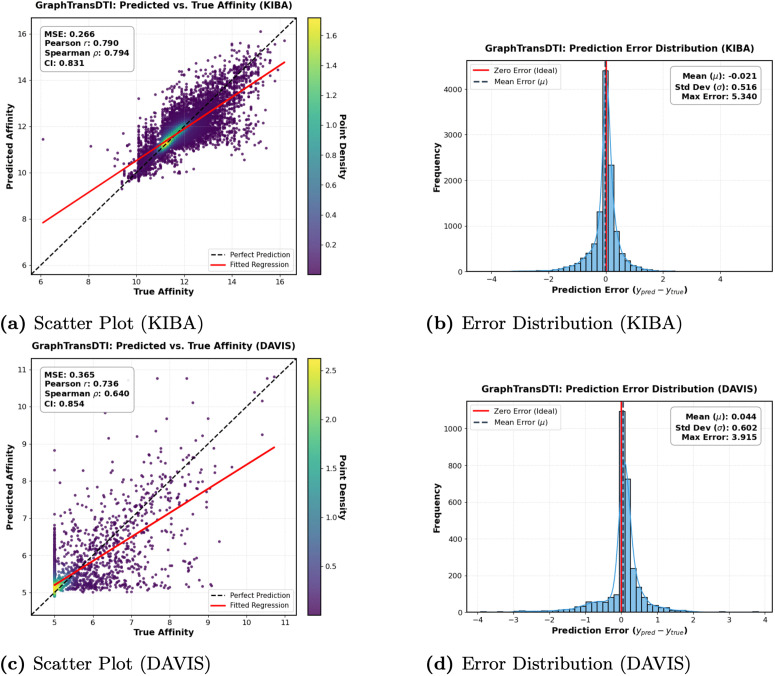
Error analysis of GraphTransDTI. The scatter plots (left) illustrate a strong correlation between predicted and experimental affinities. The error distributions (right) show near-zero mean errors (μ≈0), confirming that the model provides unbiased predictions across both datasets.

**Performance and Reliability Analysis:** The predictive power of GraphTransDTI is comprehensively evaluated through correlation and error distribution analyses. As shown in Figs 10a and 10c, the density-colored scatter plots indicate that the majority of test samples are tightly clustered along the ideal *y* = *x* line. Furthermore, the error distributions (Figs 10d and 10b) follow a Gaussian-like distribution. The extremely low mean errors (μ=−0.012 for DAVIS and μ=0.044 for KIBA) signify that the model does not suffer from systematic over-prediction or under-prediction.

**Fig 11:**
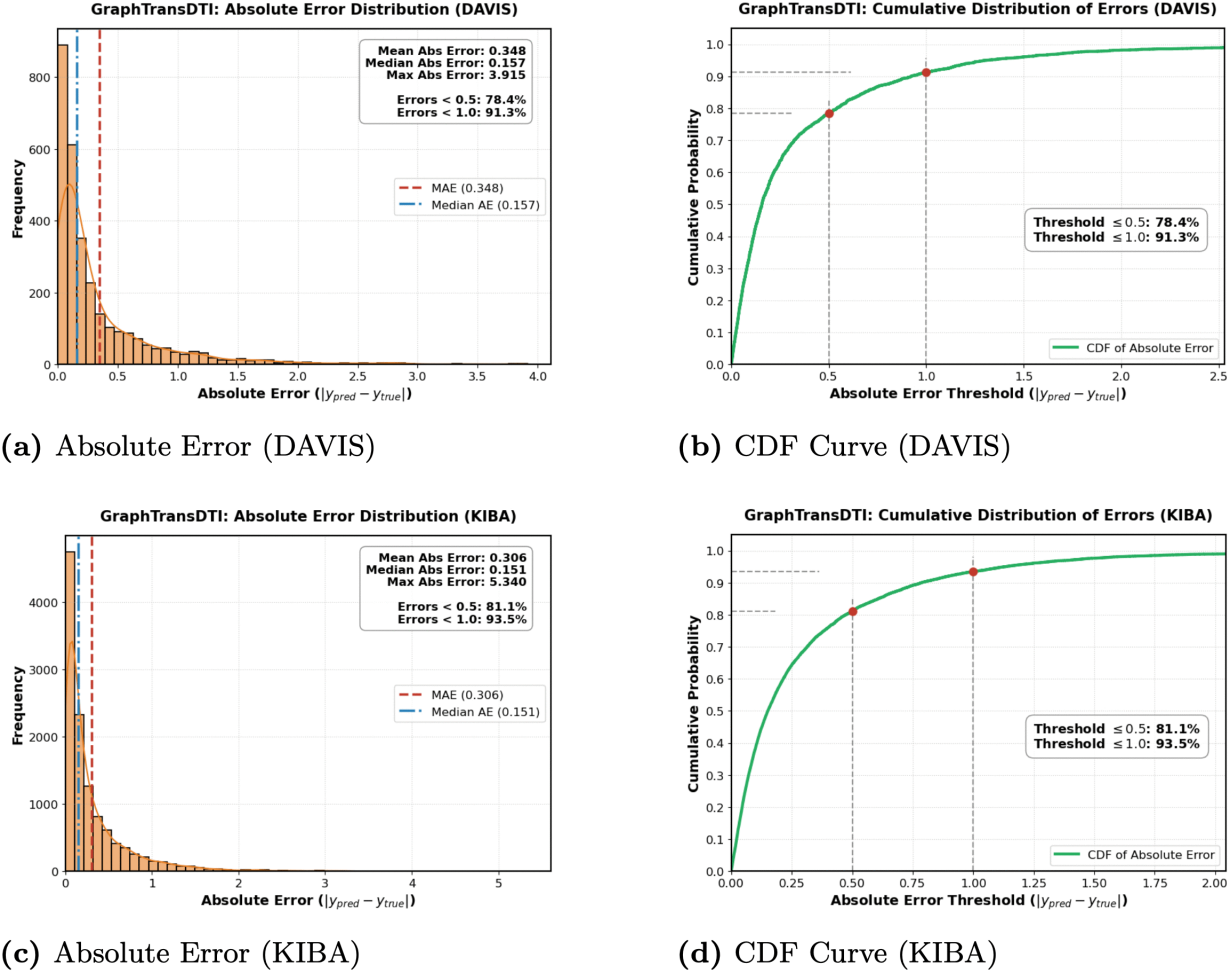
Comprehensive error magnitude analysis. The histograms (left) show the frequency of absolute deviations, while the CDF curves (right) highlight the percentage of predictions within stringent error margins.

**Error Magnitude and Cumulative Precision:** To further investigate the model’s precision, we analyzed the magnitude of absolute errors and their cumulative distribution (Fig 11). The Cumulative Distribution Function (CDF) analysis provides evidence of the model’s robustness for virtual screening. For the DAVIS dataset, 78.4% of the predictions exhibit an absolute error ≤0.5, and 91.3% fall within the ≤1.0 log unit threshold. Interestingly, despite the multi-source and complex nature of the KIBA dataset, GraphTransDTI achieves even higher cumulative precision, with 81.1% and 93.5% of predictions falling within the 0.5 and 1.0 error margins, respectively. However, the inherent noise in KIBA is reflected in its rare but extreme outliers, evidenced by a maximum absolute error of 5.340 compared to DAVIS’s strictly bounded maximum of 3.915. The heavily right-skewed nature of both absolute error histograms confirms that such significant deviations remain exceptional cases.

**Fig 12:**
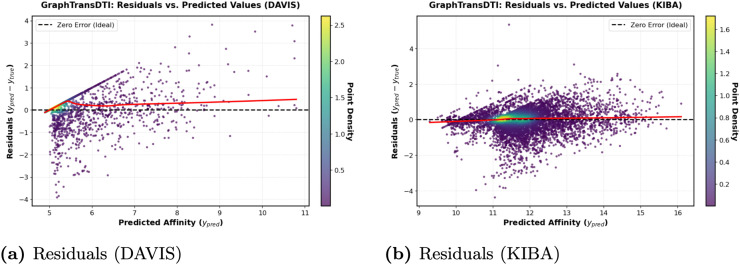
Residual plots of GraphTransDTI. The red solid line represents the LOWESS trend, confirming that the residuals are homoscedastic and independent of the predicted values.

**Residual Stability Analysis:** Finally, we performed residual analysis to detect any potential heteroscedasticity or prediction bias across different affinity ranges (Fig 12). The residual plots demonstrate that the errors are randomly distributed around the zero-baseline. Notably, the LOWESS (Locally Weighted Scatterplot Smoothing) trend lines for both datasets remain closely aligned with the zero-error axis. This stability indicates that GraphTransDTI’s accuracy is consistent regardless of the binding affinity magnitude, proving its reliability for both high-affinity inhibitor discovery and low-affinity interaction filtering.

### 5.6. Ablation studies

To rigorously verify the distinct structural contribution of each architectural component within GraphTransDTI, we conducted systematic ablation experiments. To demonstrate not only the model’s standard prediction capability but also its generalizability to novel compounds, we evaluated the framework under two distinct settings on the KIBA dataset: the standard Random Split and the highly challenging Cold Drug Split.

We assess performance using the Root Mean Square Error (RMSE) and calculate the relative percentage increase in error (ΔRMSE %) when specific functional blocks are removed. This comparative approach explicitly isolates the behavior of each module under varying degrees of task difficulty. The comprehensive results are detailed in Table 4.

**Table 4:** Ablation Study Analysis on KIBA Dataset: Random vs. Cold Drug Scenarios.

Model Variant	Random Split (Standard)		Cold Drug Split (Unseen Drugs)	
	RMSE ↓	ΔRMSE (%)	RMSE ↓	ΔRMSE (%)
**Full Model (GraphTransDTI)**	**0.5150**	–	**0.6367**	–
w/o Cross-Attention	0.5592	+8.58%	0.8140	**+27.85%**
w/o Transformer (replaced by GCN)	0.5583	+8.41%	0.6743	+5.91%
w/o BiLSTM (CNN only)	0.5448	+5.79%	0.6683	+4.96%

The results presented in Table 4 demonstrate that removing any component leads to a performance drop, confirming that all modules contribute positively to the final prediction.

Under the Random Split, the architecture exhibits a highly cohesive design. Removing the Cross-Attention (+8.58%) and the Graph Transformer (+8.41%) resulted in nearly identical performance drops, while omitting the BiLSTM also caused a noticeable degradation (+5.79%). This balanced error distribution indicates that when predicting interactions for familiar entities, all modules synergistically contribute to the final prediction.

However, the evaluation under the Cold Drug Split drastically alters this landscape. While the removal of the Graph Transformer and BiLSTM led to moderate error increases (∼5–6%), removing the Cross-Attention mechanism caused an explosive catastrophic failure, with the RMSE spiking by **27.85%**.

This massive performance gap unequivocally proves that the Cross-Attention mechanism is the fundamental engine for cold-start generalization. While standard encoders (Transformer, BiLSTM) are sufficient for extracting structural features, simple static concatenation fails when the model encounters novel chemical structures. The Cross-Attention layer acts as an essential dynamic bridge, simulating the “lock and key” biological process by actively aligning specific functional sub-structures of an unseen drug with relevant amino acid pockets. Without this dynamic alignment, the model completely loses its binding logic for novel compounds.

### 5.7. Complexity and efficiency analysis

In addition to predictive accuracy, computational efficiency is a key factor determining the practical applicability of the model. GraphTransDTI is designed with an optimal balance between deep representation capability and resource costs. Regarding space complexity, the total number of trainable parameters of the model is 2,058,049 (approximately 2.06 million). This figure is significantly lower than advanced pure Transformer-based models thereby reducing GPU memory load during training and deployment.

**Fig 13:**
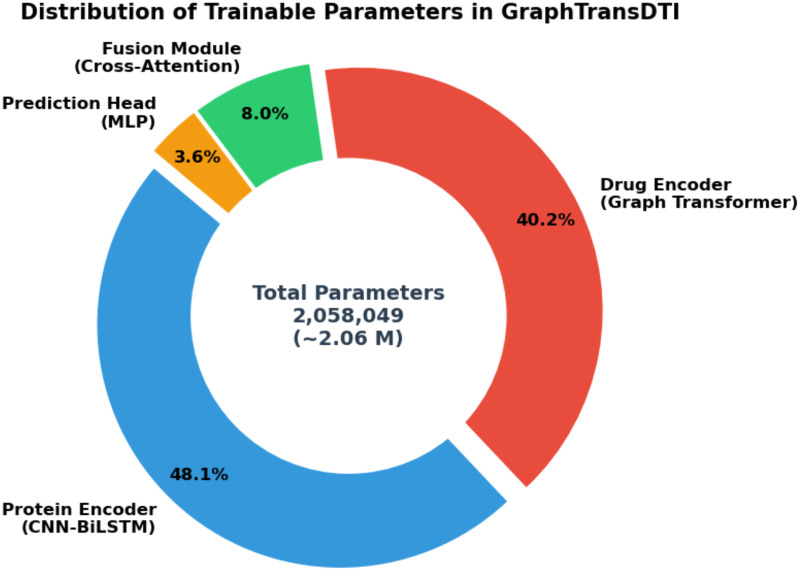
Distribution of trainable parameters across different modules of GraphTransDTI.

A detailed analysis of resource distribution, as illustrated in Fig 13, shows that the majority of parameters are concentrated in the feature encoders. Specifically, the Protein Encoder (CNN-BiLSTM) accounts for the largest proportion at 48.1% due to the requirements for multi-scale convolutional filters. The Drug Encoder (Graph Transformer) accounts for 40.2% to ensure the capacity to learn complex graph structures. Notably, the Cross-Attention Fusion module, despite occupying a modest 8.0%, plays a decisive role in improving accuracy. The remaining part is the Prediction Head (MLP), accounting for the smallest portion at 3.6%.

Regarding time complexity, the hybrid architecture helps optimize convergence speed. The use of 1D CNN layers in the initial stage of the protein encoder helps reduce local data dimensionality before feeding into the BiLSTM network, thereby minimizing sequential computation costs.

### 5.8. Discussion

Based on the comprehensive experimental evaluation across multiple benchmarks and rigorous splitting protocols, GraphTransDTI demonstrates several key architectural advantages over existing methodologies. First, the framework exhibits a strong capability for deep structural representation; the integration of the chemically-aware Graph Transformer allows the model to overcome the limitations of traditional 1D sequence-based methods like DeepDTA [4] by capturing complex and non-linear atomic interactions. Second, the model successfully exploits the multidimensional biological context of proteins. The synergistic combination of CNN and BiLSTM captures both local motifs and global sequential dependencies more effectively than single-method approaches. Third, the model facilitates bidirectional interaction alignment via the Cross-Attention mechanism. Instead of generic pooling, this mechanism operates on per-residue embeddings, allowing the model to actively focus on critical binding sites corresponding to the drug’s topological structure. Finally, compared to contemporary geometric deep learning models such as DeepTGIN [6], GraphTransDTI demonstrates competitive advantage under distribution shifts (as evidenced by its performance in cold-start scenarios) while maintaining a significantly lower computational complexity, avoiding the overhead of expensive 3D spatial convolutions.

Despite these notable advantages, the current study has certain limitations that outline directions for future research. Setting a fixed protein sequence length may lead to the loss of critical conformational information for exceptionally large macromolecules.

Regarding practical implications, GraphTransDTI consistently achieves high predictive accuracy and competitive ranking capability. By proving its stability not only on random data splits but also on unseen drugs and targets, the framework demonstrates immense potential as a reliable computational tool for drug repurposing and virtual screening. Its application promises to assist pharmaceutical researchers in confidently narrowing down the search space for novel therapeutic candidates, thereby reducing the substantial risks and costs associated with late-stage clinical trials.

## 6. Conclusion

Based on the experimental results, GraphTransDTI demonstrates an effective architectural synergy compared to traditional baselines. The model’s primary strength lies in its integrated multi-scale representation; specifically, the Graph Transformer [7] allows for the explicit capturing of long-range inter-atomic correlations, while the hybrid CNN-BiLSTM [8,9] encoder simultaneously extracts localized functional motifs and global sequential dependencies of proteins. Most critically, the Cross-Attention mechanism facilitates a dynamic “lock-and-key” interaction logic, enabling the model to bridge the gap between graph-based molecular topology and sequence-based biological semantics. This structural integration is empirically validated across two benchmark datasets, KIBA and Davis, consistently achieving competitive performance in both random and challenging cold-start scenarios (cold drug and cold target splits).

However, alongside these architectural advancements, the model retains certain limitations that require future attention. A significant constraint is the fixed protein sequence length of 1,000 characters; the necessary padding or truncation preprocessing inevitably leads to the loss of structural information for macromolecules exceeding this threshold, potentially impacting prediction accuracy for complex targets. Additionally, while the model shows improved robustness in cold-split tasks, the inherent data imbalance observed in large-scale DTI datasets poses a challenge for universal generalization.

Regarding practical implications, GraphTransDTI has proven its reliability by achieving high predictive accuracy and exceptional ranking capability, evidenced by consistent performance across the Davis and KIBA datasets. These metrics underscore the model’s immense potential for Drug Repurposing and Virtual Screening tasks. By reliably predicting binding affinities and ranking candidates even in unseen drug or target scenarios, the model serves as a powerful computational filter, helping pharmaceutical researchers significantly narrow down the search space. Consequently, this application promises to drastically reduce the risks, time, and costs associated with expensive wet-lab experiments and clinical trials.

Despite achieving promising results, there remains room for future development. The most potential direction involves integrating 3D protein structure data from the Protein Data Bank or advanced structure prediction models such as AlphaFold [29] to more accurately model spatial binding pockets. Moreover, recent advancements in Geometric Deep Learning [30] suggest that incorporating explicit 3D coordinate information could further enhance the model’s robustness against structural variations. Additionally, we plan to replace the CNN-BiLSTM encoder with Pre-trained Protein Language Models such as ESM-2 [20] or ProtBERT [31] to leverage knowledge from millions of unlabeled protein sequences. Finally, enhancing explainability through Attention Map visualization will be a key focus of future research, helping biologists understand the “why” behind the model’s predictions, specifically by identifying key atom-residue pairs involved in the binding.
